# Fatty Acid Composition and Conjugated Linoleic Acid Content in Different Carcass parts of Dağlıç Lambs

**DOI:** 10.1155/2014/821904

**Published:** 2014-01-08

**Authors:** Ali Karabacak, İbrahim Aytekin, Saim Boztepe

**Affiliations:** ^1^High Graduate School of Karapınar Aydoğanlar, University of Selçuk, 42400 Konya, Turkey; ^2^Department of Animal Science, Faculty of Agriculture, University of Selçuk, 42075 Konya, Turkey

## Abstract

This study was conducted to compare fatty acid composition and content of conjugated linoleic acid (CLA) in different regions of sheep carcasses. Lambs of the Dağlıç breed were used for this purpose. Subsequent to a 68-day period of intensive fattening, fatty acids were examined in samples taken from the legs, shoulders, breasts, and ribs of lamb carcasses. According to the analysis, in leg, shoulder, breast, and rib, respectively, total saturated fatty acids (SFA) were found to be 40.38, 42.69, 42.56, and 40.27%, unsaturated fatty acids (MUFA) were found to be 40.38, 44.17, 46.17, and 49.50%, polyunsaturated fatty acids (PUFA) were found to be 4.79, 4.29, 3.80, and 3.72%, and CLAs were found to be 1.49, 1.69, 1.53, and 1.59%.

## 1. Introduction

Meat is one of the oldest nutrients. Humankind used and benefitted from the meat of animals that they hunted and raised to meet their need for protein. With their high energy levels, fats in carcass meat have also become an indispensable nutrient. Except for fish, total meat production in the world today is 66.864.000 tonnes, with the shares of pigs, chicken, cattle, and sheep in this production being 37.01%, 30.07%, 21.04%, and 2.66%, respectively [1]. It is also certain that per capita meat consumption has decreased in recent years. In particular, the association between animal fat consumption and cardiovascular diseases (CVD) has decreased red meat consumption [2].

In studies, negative connections have been reported between saturated fats from animal sources and CVD [3–5]. Polyunsaturated fatty acid/saturated fatty acid (P/S) and *ω*6/*ω*3 ratios are highly important for human health [6]. It is accepted that lamb carcasses are fattier than other species. Therefore, customers think that less fatty meat is healthier. Nevertheless, the level of saturated fats in lamb carcass is influenced by numerous factors. Its values are close to the values of mono-unsaturated fatty acids (MUFA) and mostly below 50%. MUFA has a triglycerol-decreasing effect [7]. In addition, polyunsaturated fatty acids (PUFA) and fatty acids like *ω*3, *ω*6, *ω*7, and CLA have many advantages for human health [8–16]. For this reason, applications that would decrease the proportion of saturated fat and increase the proportion of fatty acids essential for health have been preferred [6, 17, 18]. The quantities of fatty acids in different regions of a carcass are also different. The fatty regions of a carcass are less preferred and sold for lower prices. The fatty acid content of meat is not an effective factor in pricing.

As the results of studies discussing the positive aspects of fatty acid content affecting human health become widespread, the fat content in carcass could become an effective factor in pricing carcass regions. Therefore, the current study was conducted to determine the fatty acid content of different regions in lamb carcass.

## 2. Material and Methods

In this research, 10 male lambs that were about 2.5 months old with a live weight of 20 kg on average at the outset were employed. Slaughter and cold carcass weight were determined to be 36.04 and 17.35 kg for Dağlıç. After a one-week adaptation period, Dağlıç lambs were fed ad libitum with concentrated feed and 150 g alfalfa was given to each animal for a fattening period of 68 days. The chemical composition of the concentrate feed used in the fattening period is given in Table 1. The carcass parts used to determine fatty acids were taken from the left half of the cold carcass.

At the beginning of each analysis, the samples were allowed to equilibrate to room temperature, ground, and extracted with chloroform/methanol (2 : 1 v/v) according to a standard method [19]. Methyl esters were prepared by transmethylation, using 2 mol/L KOH in methanol and n-heptane, according to method 5509 of the ISO [20].

The fatty acid methyl esters were analyzed on a HP (Hewlett Packard) Agilent 6890N model gas chromatograph (GC) equipped with a flame ionization detector (FID) and fitted with a HP-88 capillary column (100 m, 0.25 mm i.d. and 0.2 *μ*m). Chromatographic conditions were performed according to a published method [21] modified as follows: the injector and detector temperatures were 250 and 280°C, respectively. The oven was programmed at 60°C initial temperature and 1 min initial time. Thereafter the temperature was increased at 20°C/min to 190°C and held for 60 min and then increased at 1°C/min to 220°C and held for an additional 10 min at 220°C. Total run time was 107.5 min. Helium was used as the carrier gas (1 mL/min).

Identification of fatty acids and *trans* isomers was carried out by comparing sample FAME peak relative retention times with those obtained for Alltech, Nu-Check Prep. Inc. USA and Accu standards. Linoleic acid conjugated methyl ester (a mixture of *cis*- and *trans*-9,11- and -10,12-octadecadienoic acid methyl esters, catalog number O5632) was purchased from Sigma-Aldrich (St. Louis, MO, USA). Results are expressed as FID response area in relative percentages. Each reported result is the average value of three GC analyses. The results are offered as mean ± SD. The results were assessed by analysis of variance (ANOVA) at a 0.05 significance level using the minitab packet program [22]. The mean values were compared with Duncan's test.

## 3. Results and Discussion 

The fatty acid composition of different carcass regions is given in Table 2. When the results were examined, it was discovered that fatty acid compositions in the leg, shoulder, rib, and breast regions were ΣSFA 46.22, 42.69, 42.56, and 40.27%, ΣMUFA 40.38, 44.17, 46.17, and 49.50%, ΣPUFA 4.79, 4.29, 3.80, and 3.72%, ΣCLA 1.49, 1.69, 1.53, and 1.59%, Σ*ω*3 0.75, 0.53, 0.47, and 0.64%, Σ*ω*6 4.04, 3.76, 3.32, and 3.08%, and *ω*7 (palmitoleic acid C16: 1*ω*7) 3.22, 3.75, 4.14, and 4.63%. Except for ΣCLA, the differences in average fatty acid contents from the different regions were statistically significant (*P* < 0.05). ΣSFA was found to be the highest in leg region, whereas it was the lowest in the breast. While ΣMUFA was the lowest in the leg, it was the highest in the breast. The highest proportions of ΣPUFA, Σ*ω*3, and Σ*ω*6 were observed in the leg region, whereas the breast region was poor in ΣPUFA and Σ*ω*6 and the rib was the poorest region in Σ*ω*3. The highest proportion of ΣCLA was observed in the shoulder region and the lowest one was observed in the leg region. While *ω*7, which is one of the healthful fatty acids, was present in the highest proportion in the breast region (4.63), the lowest amount was in the leg (3.22). The major fatty acid within ΣSFA is palmitic acid (C16: 0) followed by stearic acid (C18: 0). The lowest levels of palmitic acid were identified in the shoulder region (22.21), while the highest proportion of palmitic acid was found in the leg region (24.46). The major fatty acid in ΣMUFA is oleic acid (C18: 1**ω**-9). This fatty acid occurred in the highest proportion in the breast region, while the lowest amount was found in the leg region (33.06). The fatty acid linoleic acid (C18: 2**ω**6) dominates among the ΣPUFA. The highest proportion of this fatty acid was observed in the leg region (3.40) and the lowest one (2.69) was seen in the breast region. The predominant fatty acid within ΣTFA is C18: 1*t*11, which had its highest value in the shoulder region (6.26) and its lowest in the breast region (3.94).

Guler and Aktumsek [23] reported the fatty acid values in omental and tail adipose tissue of Akkaraman lambs that were concentrate-fed. In that study, means for SFA were found to be higher than tail values (38.82) but less than omental values (48.95). These means are higher than all of the omental values (34.16) in our MUFA study, and the values for the shoulder and rib regions are similar to MUFA values reported for tail (45.34). The values reported for PUFA (4.74 and 4.98) are similar to our values in all regions. Our TFA values are less than both reported values (11.76 and 10.12). CLA, *ω*3, and *ω*3/*ω*6 values are higher than the values reported for omental and tail parts (0.40, 0.33, and 0.08; 0.74, 0.44, and 0.10) in each region. In this study, *ω*6 values were found to be less than the reported values (4.40 and 4.53) in all regions.

Guler et al. [24] reported fatty acid values in subcutaneous adipose tissue of Akkaraman lambs that were concentrate-fed. Our ΣSFA, ΣCLA Σ *ω*3, *ω*3/*ω*6, and *ω*7 values were all higher (41.79, 0.61, 0.41, 0.09 and 2.42), and the ΣTFA and Σ*ω*6 (10.65, 4.54) values were less than those reported by Guler et al. [24] in all regions. However, while the reported value for MUFA (41.99) is similar to what we observed in the leg region in this study, it is less than those in other regions. While the PUFA value (4.96) in the leg region shows similarity, it is higher than that in other regions in our study.

Manso et al. [25] reported the subcutaneous fatty acid composition of a control group including Merino lambs. In the current study, it was observed that the ΣSFA value is less than this value (48.15) reported for all regions. It can also be seen that ΣCLA values were higher than the findings of Manso et al. [25] (0.46). While the reported value (47.22) for ΣMUFA by Manso et al. [25] is similar to the values for the rib and breast regions in our study, it is higher than the values for the leg and shoulder regions. On the other hand, the PUFA (4.63) value of Manso et al. is similar to the values for the leg and shoulder regions from our study, while it is higher than the values for the rib and breast regions.

Díaz et al. [26] reported SFA, MUFA, and PUFA values in subcutaneous fat from lambs fed indoors as 57.16, 37.63 and 5.21% in the loin region, and 56.80, 35.22, and 5.12% in the leg region, respectively. The ΣSFA and ΣPUFA values obtained in this study are less than these reported values, and the ΣMUFA value is higher than the reported value.

Sañudo et al. [27] reported intramuscular fatty acid values in Spanish Merino, Rasa Aragonesa, and Welsh Mountain breeds. Our palmitoleic acid fatty acid values in all regions were higher than those reported values (2.69, 2.66, and 2.12, resp.). The SFA value for Spanish Merino (43.22) is similar to the shoulder and rib values in our study. The SFA value for Rasa Aragonesa (47.35) is similar to that of the leg region in our study. On the other hand, the SFA value for Welsh Mountain (49.97) is higher than the corresponding values for all regions in our study. Our PUFA value was less than the values (14.61, 15.84, and 6.99) reported for the three former breeds.

Rowe et al. [28] reported SFA, MUFA, and PUFA values (49.36, 40.68, and 4.74, resp.) for lamb fed in a drylot system. Compared to Rowe et al., the SFA values in this study are lower than the values reported for each region. MUFA values are similar in the leg region but higher than the values reported in other regions. Once again, the PUFA value shows similarity in the leg region but is lower in other regions.

Castro et al. [29] reported myristic acid (C14: 0 (4.73)), palmitic acid (24.64), margaric acid (C17: 0 (2.51)), oleic acid (10.36), palmitoleic acid (2.09), linoleic acid (42.32), linolenic acid (4.36), SFA (42.75), MUFA (45.98), and PUFA (4.83) averages for subcutaneous fat in a control group consisting of Ojalada lambs. The value reported by Castro et al. [29] for myristic acid is higher than that found in all regions in this study. On the other hand, the value of palmitic acid is similar to the values in our study. The reported value for margaric acid is similar to the breast region, and the reported value of stearic acid is higher than the shoulder, rib and breast values in the present study. While the reported value for palmitoleic acid is lower than all regions in our study, it can be seen that values for oleic acid and linoleic acid are higher than all regions in our study. The reported values for SFA and MUFA are similar to the shoulder and rib values in this study, while the PUFA value is similar to our leg value.

Mir et al. [30] reported on palmitic acid (27.5 and 30.0), stearic acid (14.7 and 17.4), oleic acid (47.9 and 45.5), linoleic acid (8.6 and 5.7), and linolenic acid (1.1 and 1.3) values in the leg and rib regions of Suffolk X Dorset crossbred lambs in the control group. Our findings were found to be less than the values reported by Mir et al. [30], yet the breast region values are similar to the values for oleic acid in their study.

Bas and Morand-Fehr [31] indicated the myristic acid (4.0 and 5.0), palmitic acid (24.4 and 23.9), margaric acid (2.7 and 1.6), stearic acid (15.6 and 14.4), palmitoleic acid (3.2 and 3.0), oleic acid (42.5 and 43.8), and linoleic acid (3.4 and 3.6) values in rib and leg in their study of lamb carcasses. Compared to these reported values, myristic acid values exhibit similarity to the leg and breast values of this study. Palmitic acid values are similar to those from all the regions. The reported margaric acid value is close to the value in the breast region. The reported stearic acid value is higher than the values in all regions of our study. The reported oleic acid value shows similarity to the value in the leg region, while the linoleic acid value shows similarity to the value for the rib region and the linolenic acid value is similar to the values in the leg, rib, and shoulder regions.

## 4. Conclusions

Consequently, when fatty acid content in carcass regions of Dağlıç lambs is examined, it can be seen that the breast region has the lowest values in terms of SFA and the highest values in terms of MUFA. The lowest proportion of total TFA is observed in the breast region. In addition, the breast region comes to the forefront in terms of the *ω*3/*ω*6 ratio. In light of these data, it is possible to say that the fatty acid content of the breast region is more advantageous than that in other carcass regions. Meat from this region can be recommended to consumers.

## Figures and Tables

**Table 1:** Chemical composition of the concentrate feed (%).

Chemical composition	Percentage (%)
Dry matter	88.00
Organic matter	78.09
Crude protein	14.52
Crude fiber	9.89
Ash	9.91
Crude fat	1.59
Calcium	0.60
Phosphorus	0.40
Calculated metabolizable energy (kcal/kg)	2562

**Table 2:** Fatty acid composition of different carcass parts of Dağlıç (g/100 g total fatty acids).

Fatty acid	Leg	Shoulder	Rib	Breast
C 10 : 0	0.18 ± 0.06	0.21 ± 0.08	0.19 ± 0.08	0.19 ± 0.05
C 11 : 0*	0.05 ± 0.02^bc^	0.11 ± 0.05^a^	0.08 ± 0.04^ab^	0.04 ± 0.03^c^
C 12 : 0	0.22 ± 0.04^b^	0.36 ± 0.12^a^	0.43 ± 0.17^a^	0.30 ± 0.10^ab^
C 13 : 0	0.08 ± 0.03^b^	0.11 ± 0.03^ab^	0.13 ± 0.04^a^	0.08 ± 0.04^b^
C 14 : 0	3.99 ± 0.52^ab^	3.24 ± 0.63^bc^	2.96 ± 0.58^c^	4.50 ± 0.90^a^
C 15 : 0	1.63 ± 0.45^bc^	1.98 ± 0.34^ab^	2.22 ± 0.54^a^	1.38 ± 0.41^c^
C 16 : 0	24.46 ± 1.82	22.21 ± 2.10	22.63 ± 1.92	22.96 ± 2.02
C 17 : 0	3.32 ± 0.23^b^	4.20 ± 0.53^a^	4.56 ± 0.37^a^	2.50 ± 0.56^c^
C 18 : 0	11.83 ± 2.18^a^	9.87 ± 1.43^ab^	8.97 ± 2.11^b^	7.87 ± 1.22^b^
C 19 : 0	0.22 ± 0.03^b^	0.24 ± 0.03^b^	0.20 ± 0.03^b^	0.31 ± 0.04^a^
C 20 : 0	0.11 ± 0.09	0.08 ± 0.07	0.11 ± 0.07	0.06 ± 0.03
C 21 : 0	0.11 ± 0.07	0.07 ± 0.04	0.07 ± 0.04	0.07 ± 0.02
C 22 : 0	0.02 ± 0.02^a^	0.01 ± 0.00^b^	0.01 ± 0.00^b^	0.01 ± 0.01^b^
**Σ SFA **	**46.22 ± 2.20** ^ a^	**42.69 ± 2.90** ^ b^	**42.56 ± 2.60** ^ b^	**40.27 ± 3.49** ^ b^
C 14 : 1*ω*5	0.32 ± 0.04^b^	0.38 ± 0.05^ab^	0.41 ± 0.09^a^	0.37 ± 0.07^ab^
C 15 : 1*ω*5	0.37 ± 0.07^b^	0.54 ± 0.08^a^	0.56 ± 0.15^a^	0.36 ± 0.09^b^
C 16 : 1*ω*7	3.22 ± 0.42^c^	3.75 ± 0.32^bc^	4.14 ± 0.44^ab^	4.63 ± 0.56^a^
C 17 : 1*ω*8	1.88 ± 0.35^c^	2.87 ± 0.68^b^	3.67 ± 0.77^a^	2.55 ± 0.66^bc^
C 18 : 1*ω*9	33.06 ± 2.52^b^	35.01 ± 1.67^b^	35.86 ± 1.80^b^	39.92 ± 3.38^a^
C 18 : 1*ω*7	1.42 ± 0.15	1.53 ± 0.16	1.44 ± 0.15	1.58 ± 0.21^a^
C 20 : 1*ω*9	0.09 ± 0.07	0.08 ± 0.03	0.08 ± 0.03	0.08 ± 0.03
C 22 : 1*ω*9	0.02 ± 0.01	0.01 ± 0.00	0.01 ± 0.00	0.01 ± 0.00
**Σ MUFA **	**40.38 ± 2.34** ^ c^	**44.17 ± 2.39** ^ b^	**46.17 ± 2.20** ^ ab^	**49.50 ± 4.16** ^ a^
C 18 : 2*ω*6	3.40 ± 0.91	3.33 ± 0.62	2.90 ± 0.55	2.69 ± 0.41
C 18 : 3*ω*6	0.19 ± 0.13^a^	0.13 ± 0.07^ab^	0.08 ± 0.04^b^	0.10 ± 0.03^b^
C 18 : 3*ω*3	0.45 ± 0.14^ab^	0.41 ± 0.10^ab^	0.32 ± 0.08^b^	0.49 ± 0.11^a^
C 20 : 2*ω*6	0.12 ± 0.07^a^	0.09 ± 0.03^ab^	0.11 ± 0.04^a^	0.07 ± 0.01^b^
C 20 : 3*ω*6	0.07 ± 0.04^a^	0.05 ± 0.01^b^	0.04 ± 0.01^b^	0.04 ± 0.01^b^
C 20 : 3*ω*3	0.03 ± 0.03	0.01 ± 0.00	0.01 ± 0.01	0.01 ± 0.01
C 20 : 4*ω*6	0.14 ± 0.05	0.10 ± 0.03	0.11 ± 0.06	0.12 ± 0.03
C 20 : 5*ω*3	0.06 ± 0.06^a^	0.02 ± 0.02^b^	0.03 ± 0.02^b^	0.03 ± 0.01^b^
C 22 : 2*ω*6	0.04 ± 0.06	0.01 ± 0.00	0.01 ± 0.01	0.01 ± 0.01
C 22 : 3*ω*3	0.07 ± 0.05^a^	0.02 ± 0.01^b^	0.05 ± 0.06^ab^	0.02 ± 0.02^b^
C 22 : 4*ω*6	0.05 ± 0.04	0.04 ± 0.01	0.05 ± 0.03	0.03 ± 0.02
C 22 : 5*ω*6	0.03 ± 0.02^a^	0.01 ± 0.00^a^	0.02 ± 0.01^b^	0.02 ± 0.01^b^
C 22 : 5*ω*3	0.10 ± 0.07^a^	0.05 ± 0.01^b^	0.05 ± 0.02^b^	0.07 ± 0.02^ab^
C 22 : 6*ω*3	0.04 ± 0.03^a^	0.02 ± 0.01^b^	0.02 ± 0.01^ab^	0.02 ± 0.01^b^
**Σ PUFA **	**4.79 ± 1.37** ^ a^	**4.29 ± 0.74** ^ ab^	**3.80 ± 0.65** ^ b^	**3.72 ± 0.50** ^ b^
C 14 : 1*t*9	0.10 ± 0.03^b^	0.14 ± 0.03^ab^	0.18 ± 0.04^a^	0.19 ± 0.07^a^
C 16 : 1*t*9	0.47 ± 0.05^a^	0.44 ± 0.09^a^	0.34 ± 0.08^b^	0.49 ± 0.05^a^
C 18 : 1*t*9	0.08 ± 0.02^b^	0.12 ± 0.04^a^	0.13 ± 0.03^a^	0.09 ± 0.06^ab^
C 18 : 1*t*11	6.24 ± 1.01^a^	6.26 ± 1.66^a^	5.11 ± 1.43^ab^	3.94 ± 0.87^b^
C 18 : 2*t*9,*t*12	0.12 ± 0.02^a^	0.09 ± 0.03^ab^	0.08 ± 0.03^b^	0.09 ± 0.02^b^
C 18 : 2*t*9,*c*12	0.11 ± 0.02	0.11 ± 0.04	0.10 ± 0.04	0.12 ± 0.03
**Σ TFA **	**7.12 ± 1.05** ^ a^	**7.16 ± 1.72** ^ a^	**5.94 ± 1.54** ^ ab^	**4.92 ± 0.89** ^ b^
C 18 : 2 *c*9,*t*11	1.45 ± 0.19	1.67 ± 0.29	1.51 ± 0.35	1.57 ± 0.24
C 18 : 2 *t*10,*c*12	0.02 ± 0.01	0.01 ± 0.00	0.01 ± 0.01	0.01 ± 0.00
C 18 : 2 *c*11,*t*13	0.02 ± 0.01^a^	0.01 ± 0.00^b^	0.01 ± 0.00^b^	0.01 ± 0.00^b^
**ΣCLA **	**1.49 ± 0.20 **	**1.69 ± 0.29 **	**1.53 ± 0.35 **	**1.59 ± 0.25 **
**Σ *ω*3**	**0.75 ± 0.27** ^ a^	**0.53 ± 0.11** ^ ab^	**0.47 ± 0.14** ^ b^	**0.64 ± 0.09** ^ ab^
**Σ *ω*6**	**4.04 ± 1.15** ^ a^	**3.76 ± 0.66** ^ ab^	**3.32 ± 0.57** ^ b^	**3.08 ± 0.44** ^ b^
***ω*3/*ω*6**	**0.19 ± 0.04** ^ ab^	**0.14 ± 0.02** ^ b^	**0.14 ± 0.04** ^ b^	**0.21 ± 0.03** ^ a^

^∗a–c^The difference among averages indicated by different letters in the same line is significant (*P* < 0.05).
